# Value of Preoperative Imaging Results in Predicting Cochlear Nerve Function in Children Diagnosed With Cochlear Nerve Aplasia Based on Imaging Results

**DOI:** 10.3389/fnins.2022.905244

**Published:** 2022-06-14

**Authors:** Xiuhua Chao, Ruijie Wang, Jianfen Luo, Haibo Wang, Zhaomin Fan, Lei Xu

**Affiliations:** Department of Otolaryngology-Head and Neck Surgery, Shandong Provincial ENT Hospital, Shandong University, Jinan, China

**Keywords:** cochlear nerve deficiency, cochlear implantation, imaging, electrically evoked compound action potential, cochlear nerve aplasia

## Abstract

This study aimed to assess the function of the cochlear nerve using electrically evoked compound action potentials (ECAPs) for children with cochlear implants who were diagnosed with cochlear nerve aplasia and to analyze the correlation between preimplantation imaging results and ECAP responses. Thirty-five children diagnosed with cochlear nerve aplasia based on magnetic resonance imaging (MRI) were included. Preimplantation MRI and high-resolution computed tomography (HRCT) images were reconstructed, and the width of the bone cochlear nerve canal (BCNC), the diameter of the vestibulocochlear nerve (VCN), and the diameter of the facial nerve (FN) were measured. ECAP input/output (I/O) functions were measured at three electrode locations along the electrode array for each participant. The relationship between ECAP responses (including ECAP threshold, ECAP maximum amplitude, and slope of ECAP I/O function) and sizes of the BCNC and VCN was analyzed using Pearson's correlation coefficients. Our analysis revealed that ECAP responses varied greatly among individual participants. Overall, ECAP thresholds gradually increased, while maximum amplitudes and ECAP I/O function slopes gradually decreased, as the electrode location moved from the basal to the apical direction in the cochlea. ECAP responses exhibited no significant correlations with BCNC width or VCN diameter. The ratio of the VCN to FN diameters was significantly correlated with the slope of the ECAP I/O function and the maximum amplitude. BCNC width could not predict the function of the cochlear nerve. Compared with the absolute size of the VCN, the size of the VCN relative to the FN may represent an indicator for predicting the functional status of the cochlear nerve in children diagnosed with cochlear nerve aplasia based on imaging results.

## Introduction

Cochlear nerve aplasia or hypoplasia is defined as an absent (aplasia) or a small (hypoplasia) cochlear nerve based on the results of magnetic resonance imaging (MRI). Clinically, children with deficient cochlear nerves often exhibit severe-to-profound sensorineural hearing loss (SNHL). The main treatment for hearing reconstruction in these children is cochlear implantation. However, children with cochlear nerve deficiency (CND) have poor outcomes after cochlear implantation. Previous studies reported that the benefits of cochlear implants (CIs) in patients with CND were worse than in other children with SNHL who had normal-sized cochlear nerves and varied greatly among individual children (Ehrmann-Muller et al., 2018; Arumugam et al., 2020). Although only a few patients can achieve simple open-set speech perception skills, most patients only exhibit improvements in sound awareness, and a few patients may experience no benefits following implantation (Kang et al., 2010; Kutz et al., 2011; Young et al., 2012; Vincenti et al., 2014). Unfortunately, to date, there are no effective methods for predicting the benefits of CIs preoperatively. Thus, providing appropriate counseling regarding the outcomes of cochlear implantation for children with CND remains challenging, as does determining the optimal ear in cases of unilateral implantation for children with bilateral CND.

Currently, the diagnosis of CND mainly depends on imaging findings. CND is diagnosed if the cochlear nerve is absent or smaller than the adjunct facial nerve (FN) in the internal auditory canal on MRI (Casselman et al., 1997; Sennaroglu and Bajin, 2017). In addition, temporal bone high-resolution computed tomography (HRCT) can be helpful for assessing the health of the cochlear nerve. The cochlear nerve is considered hypoplastic or aplastic when the diameter of the bony cochlear nerve canal (BCNC) is <1.5 mm and the diameter of the internal auditory canal is <2 mm (Miyasaka et al., 2010; Yan et al., 2013). The electrically evoked auditory brainstem response to the application of an intracochlear testing electrode is also an important indicator of the integrity of the cochlear nerve (Lassaletta et al., 2017). However, this assessment is somewhat invasive and traumatic and requires anesthesia. Previous studies have demonstrated that the morphology of the cochlear nerve or vestibulocochlear nerve (VCN) and the width of the BCNC might predict the degree of CND. Several studies have demonstrated that patients with aplastic cochlear nerves tend to perform worse than those with hypoplastic cochlear nerves following cochlear implantation (Kutz et al., 2011; Wu et al., 2015; Peng et al., 2017). Additionally, the width of the BCNC is positively correlated with the diameter of the cochlear nerve, and a narrower BCNC has been associated with more severe hearing loss and lower speech discrimination scores than a wider BCNC (Purcell et al., 2015). Thus, the size of the cochlear nerve or VCN and the width of the BCNC on imaging might be indicators of the severity of CND. However, conflicting results have been reported in previous studies regarding the relationship between preoperative imaging results and postoperative CI outcomes for children with CND. Although some studies have reported better auditory performance in children with normal BCNC than in those with BCNC stenosis (Chung et al., 2018; Kang et al., 2019), other studies have not revealed any predictive value of BCNC width for auditory or speech performance in children with CND (Warren et al., 2010; Tahir et al., 2020). In addition, the results of some studies have indicated an association between a larger VCN size in relation to the FN and better CI outcomes (Yamazaki et al., 2015; Han et al., 2019), while others have found no correlation between the size of the cochlear nerve or VCN and postimplantation auditory performance (Chao et al., 2016; Jain et al., 2020).

Although the health of the cochlear nerve is a critical factor affecting the postoperative effects of cochlear implantation, implantation age, history of hearing aid use, cognitive ability, parental socioeconomic status, and language training are also factors contributing to the outcomes of cochlear implantation. Thus, the relationships between preoperative imaging results and cochlear nerve function remain unclear. A better understanding of the value of preoperative imaging in predicting the degree of the cochlear nerve lesion could enable us to better assess CI candidacy and provide appropriate patient counseling for the benefits of CI for individual children with deficient cochlear nerves.

Recently, electrically evoked compound action potentials (ECAPs) have been widely used to evaluate cochlear nerve function in patients with implants (He et al., 2017). The ECAP response is generated by a group of auditory nerve fibers that are activated by electrical stimuli. It could be recorded using the “reverse” telemetry function implemented in current CI devices. Previous studies have shown that the slope of the ECAP input/output (I/O) function and the ECAP amplitude evoked by the most comfortable level are associated with the density of the surviving neural population, with steeper slopes and larger amplitudes suggesting a larger number of residual neurons (Miller et al., 2008; Pfingst et al., 2015). In addition, the ECAP threshold, which refers to the lowest stimulation level that could evoke an ECAP response, may also reflect the neural population to some extent (Ramekers et al., 2014). Thus, the aims of this study were to assess the function of the cochlear nerve using ECAP responses for individual children diagnosed with cochlear nerve aplasia based on imaging results and to analyze the correlation between imaging results (width of the BCNC and size of the VCN) and ECAP responses.

## Materials and Methods

### Ethics Statements

This study was approved by the Ethical Committee of Shandong Provincial ENT Hospital affiliated with Shandong University (No. XYK20170906). Informed consent was obtained from the legal guardians of participants prior to participation.

### Study Design and Population

This cohort study included 35 children (CND1–CND35; 11 boys and 24 girls), with cochlear nerve aplasia diagnosed by MRI. All participants were implanted with the Cochlear® Nucleus device (Cochlear Ltd.) in one or both ears. Children were included only if they had raw HRCT and MRI data that could be reconstructed and reanalyzed.

### Radiological Assessment

**Figure 1:**
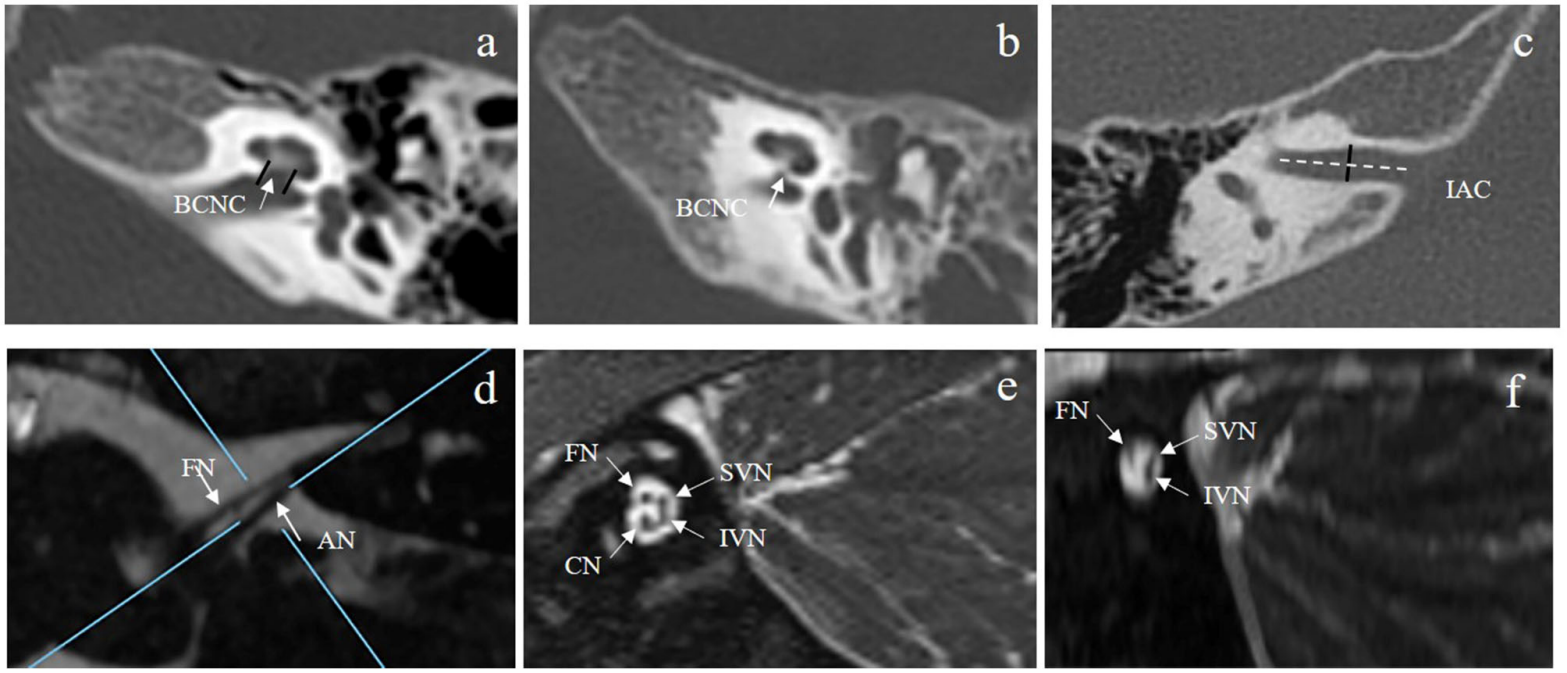
**(A,B)** show the normal BCNC at the mid-modiolar level on the axial plane of HRCT **(A)** (two short black lines and white arrow) and a case of BCNC stenosis **(B)**. **(C)** shows the IAC diameter measured at the middle of the IAC on the axial plane of HRCT (short black line). **(D)** shows the vestibulocochlear nerve and the facial nerve at the cerebellopontine angle on the axial plane of MRI. The blue lines illustrate the plane prescribed for oblique plane sagittal images obtained perpendicular to the nerves of the IAC. **(E)** shows the cochlear, facial, superior vestibular, and inferior vestibular nerves on a reconstructed image in a patient with a normal cochlear nerve. **(F)** shows that the cochlear nerve could not be observed for one participant with cochlear nerve aplasia. BCNC, bone cochlear nerve canal; HRCT, high-resolution computed tomography; IAC, inner auditory canal.

All participants underwent MRI and HRCT for evaluation of the cochlear nerve status and other inner ear malformations before the operation in accordance with the previously described protocols (Chao et al., 2016). HRCT was performed using a 64-slice multidetector CT scanner (Somatom Sensation Cardiac 64; Siemens, Munich, Germany) using a standard temporal bone protocol. The main parameters for HRCT were as follows: the tube voltage was 120 kV; the tube current was automated tube current modulation (CareDose4D, Siemens); the slice thickness was 0.6 mm; the window width and window level were 4,000 HU and 600 HU, respectively. Axial and coronal images were obtained. Then, the axial images were reconstructed parallel to the lateral semicircular canal in a standard plane. The BCNC was evaluated between the anterior lower part of the bottom of the inner ear canal and the cochlear axis on axial HRCT, and the width of the BCNC was measured in the middle of the BCNC (Figure 1A). BCNC stenosis was diagnosed when BCNC width was <1.5 mm (Figure 1B), and the absence of the BCNC on any plane on HRCT was defined as atresia (Purcell et al., 2015; Lim et al., 2018). The width of the midportion of the inner auditory canal (IAC) was also measured at the level of the porus acusticus, from its posterior margin to the anterior wall of the IAC along a line orthogonal to the long axis of the IAC (Figure 1C) (Purcell et al., 2015). IAC stenosis was defined as an IAC width <3 mm. In addition, the structure of the inner ear was evaluated for any malformations.

Magnetic resonance imaging was performed using a clinical 3.0T MRI system (MAGNETOM Verio; Siemens) equipped with a 64-channel array head and neck coil. MRI sequences included axial T1-weighted and T2-weighted (T2W) imaging and three-dimensional fast spin-echo T2W sequences. Cochlear nerves and VCNs were evaluated on T2W axial and three-dimensional fast spin-echo T2-weighted sequences. The main parameters for the T2W sequences were as follows: the field of view was 162 × 82 mm; the repetition time was 1,200 ms; the echo time was 125 ms; the image matrix was 320 × 164; and the slice thickness was 0.5 mm. The main parameters for the three-dimensional fast spin-echo T2W sequences were as follows: the field of view was 220 × 220 mm; the repetition time was 1,200 ms; the echo time was 129 ms; the image matrix was 320 × 164; and the slice thickness was 0.2 mm. The diameters of the VCN and FN were measured at the cerebellopontine angle (Figure 1D), and the ratio of the VCN diameter to the FN diameter (VCN/FN ratio) was calculated. The VCN was characterized as hypoplastic if the nerve diameter was <1.5 mm or smaller than the FN with normal function (Sennaroglu, 2010). Direct oblique sagittal images perpendicular to the long axis of the IAC were reconstructed to show the cochlear, facial, superior vestibular, and inferior vestibular nerves in the IAC (Figure 1E). Cochlear nerve hypoplasia was diagnosed if the diameter of the cochlear nerve was smaller than that of the adjacent FN or the cochlear nerve in the contralateral ear. Cochlear nerve aplasia was diagnosed if the cochlear nerve could not be identified on any plane of the MRI (Figure 1F). All imaging results were reviewed by two experienced radiologists and an otologist. The results were defined as the average values measured by three persons.

### Measurement of ECAPs

**Figure 2:**
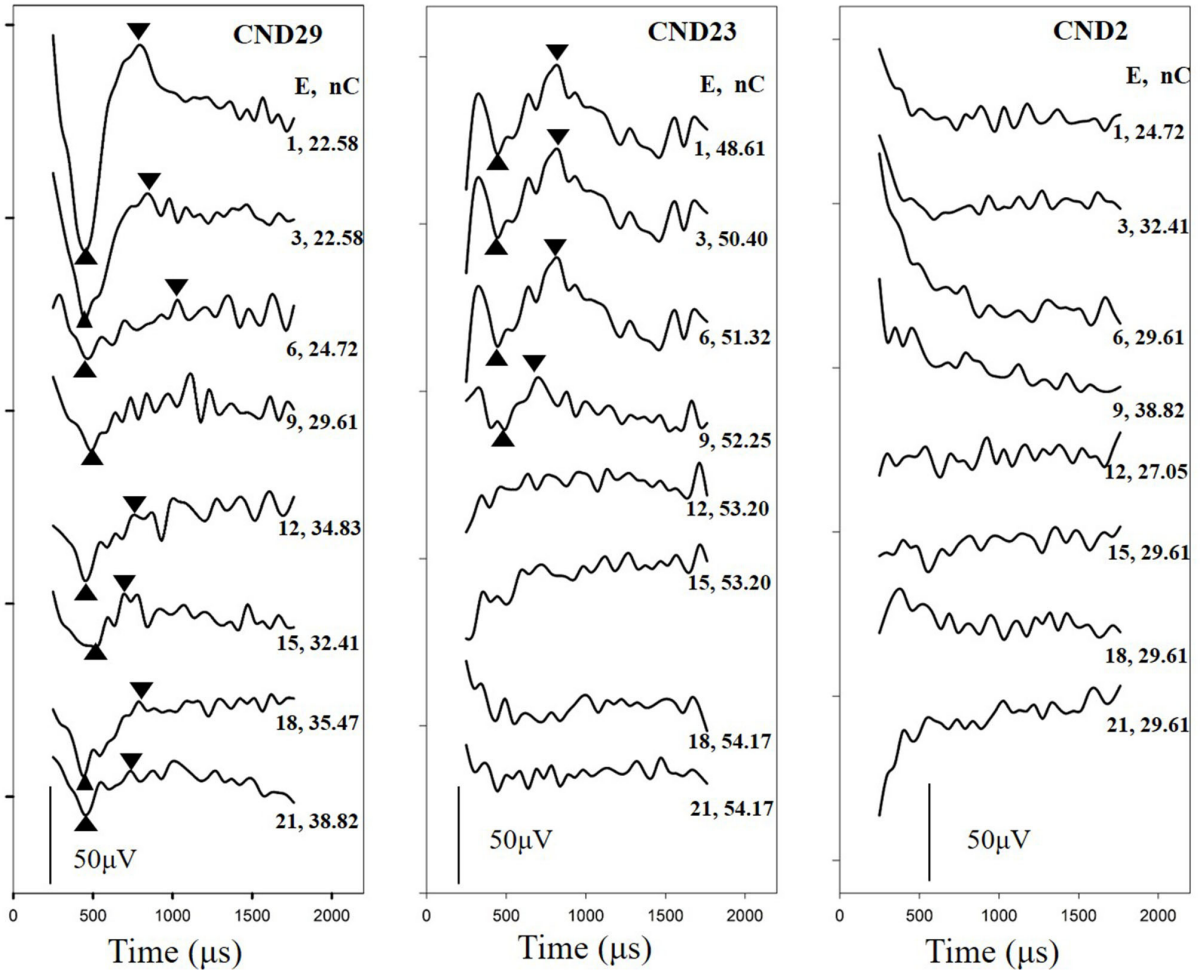
Waveforms of ECAP responses were recorded from multiple electrodes in three children with cochlear nerve aplasia diagnosed based on MRI images. For CND29, the ECAP was recorded from all electrodes; for CND23, the ECAP was recorded from electrodes 1–9; and for CND2, the ECAP could not be recorded from any electrodes. Tested electrodes and stimulation levels are shown on the right of each panel. E, tested electrode; nC, stimulation level; ECAP, electrically evoked compound action potential.

Electrically evoked compound action potentials were measured using the advanced neural response telemetry function implanted in the Custom Sound EP (version 4.3) software (Cochlear Ltd., Sydney, Australia). For each participant, the maximum comfort level was tested for each electrode before the ECAP recording. This level was defined as the largest stimulation level at which the participants felt comfortable. For children who could not provide a behavioral response, this level was defined as the largest stimulation level that would not cause discomfort. Two pulse forward masking methods were used to record the ECAP waveforms in this study. The stimulation and recording parameters used to record the ECAP were selected according to a previously described protocol (He et al., 2020). The stimulus was a single cathodic-leading biphasic charge-balanced pulse. The masker-to-probe interval was 400 μs, the probe rate was 15 Hz, the pulse width varied across individuals from 37 to 75 μs/phase, and the inter-phase gap was 7 μs. The recording electrode was placed two or three electrodes away from the stimulating electrode in the basal direction with a sampling delay of 98–142 μs. These parameters were adjusted for each participant to minimize artifacts and obtain optimized ECAP morphologies. First, ECAP responses were recorded from each electrode along the electrode array. Second, the ECAP I/O function was measured at three electrode locations where the ECAP waveforms could be recorded. Electrodes 3, 12, and 21 were selected for participants whose ECAPs could be recorded at all electrode locations. For participants in whom ECAPs could only be recorded at some electrode locations, the selected electrodes were extended to the most apical electrode location with a measurable ECAP, and the testing electrodes were equally separated. The selected electrodes were defined as the basal, middle, and apical electrodes in this study. Figure 2 shows the waveforms of the ECAP response from multiple electrodes for three participants. For CND29, the ECAP was recorded from all electrodes; for CND23, ECAPs were recorded from electrodes 1–9; and for CND2, the ECAP could not be recorded from any electrodes.

For the ECAP I/O function, the probe level was started at the C level and decreased in steps of two to three current levels (CLs) until no response could be visually identified and was subsequently increased in steps of 1 CL until five continuous ECAPs were measured. The ECAP threshold was defined as the lowest stimulation level that could evoke an ECAP with an amplitude ≥5 μV. Another five continuous ECAP traces below the ECAP thresholds were tested using a step size of 1 CL. For each participant, it took approximately 2–3 h to collect all the ECAP threshold data.

### Data and Statistical Analyses

**Figure 3:**
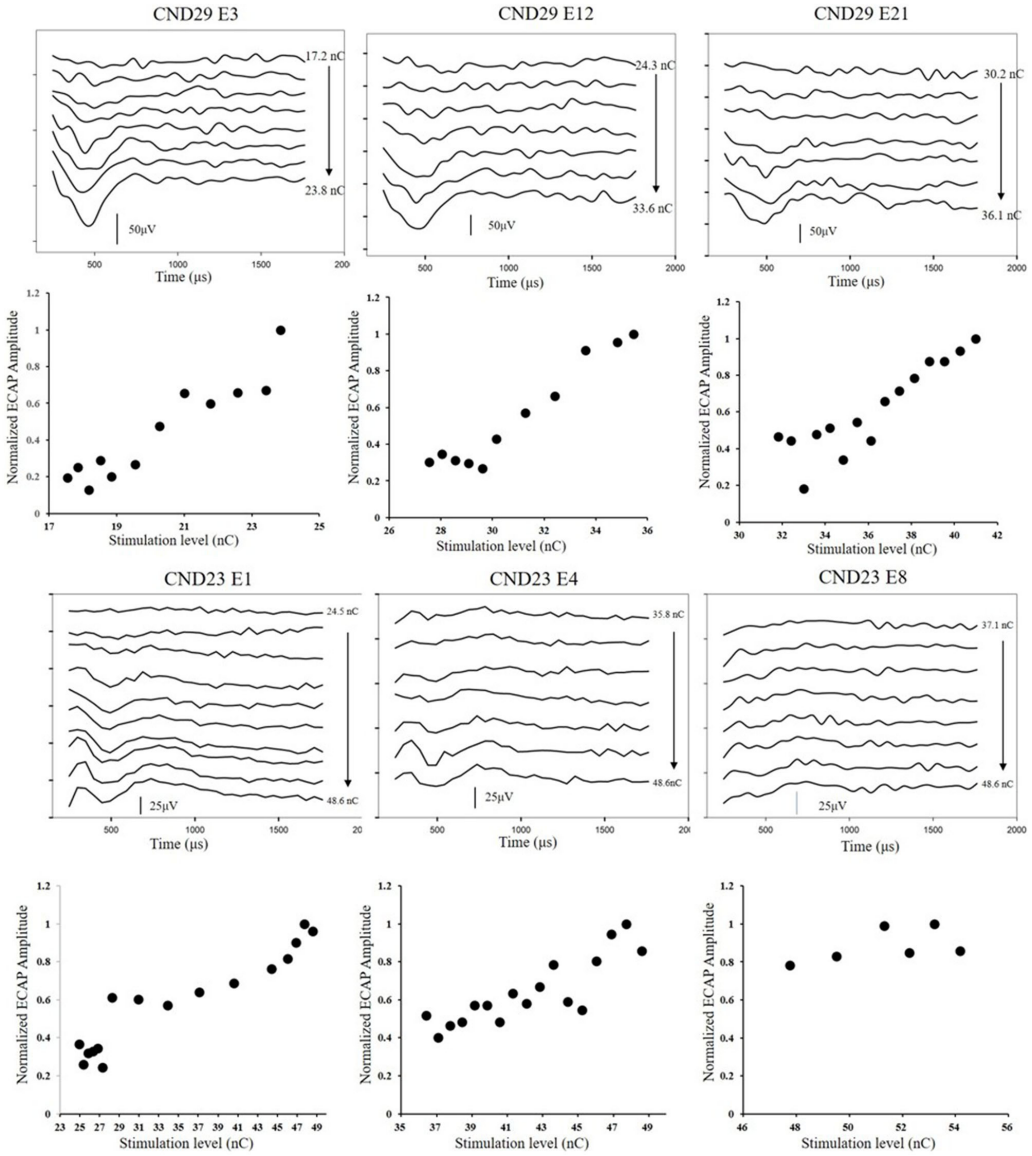
ECAP response traces and ECAP I/O functions measured at three electrode locations in CND29 (upper) and CND23 (lower). Participants and electrode numbers are displayed in each panel. The round dots represent normalized ECAP amplitudes measured at different stimulation levels. E, electrode; ECAP, electrically evoked compound action potential; CND, cochlear nerve deficiency; I/O, input/output.

All ECAP thresholds were determined based on a mutual agreement between two audiologists who reviewed the data independently. As the pulse widths used among the participants were different, ECAP thresholds were converted to units of electrical charge per phase (nC). The ECAP amplitude was defined as the difference in the amplitude between the N1 and P2 peaks of the response. The slope of the ECAP I/O function was estimated using a sigmoidal regression function, as illustrated in previous studies (He et al., 2018). Figure 3 displays ECAP response traces and ECAP I/O functions measured at three electrode locations in CND29 (upper) and CND23 (lower). ECAP amplitudes were normalized to the ECAP response tested at the maximum stimulation level. For both participants, ECAP thresholds gradually increased, while the maximum amplitudes gradually decreased, from basal to apical electrode sites. In this study, we measured ECAP responses that can represent cochlear nerve function, including the ECAP threshold, ECAP maximum amplitude, and slope of the ECAP I/O function.

The ECAP results tested at different electrode locations within participants were compared using the repeated-measures analyses of variance (ANOVA) test. In this study, the ECAP threshold, maximum ECAP amplitude, and slope of the ECAP I/O function for individual participants were defined as the mean values of the results tested at the three electrodes along the electrode array. The correlations between ECAP responses and radiological findings for all participants were analyzed using the Pearson correlation test. All statistical analyses were conducted using SPSS Statistics 21 (IBM Corp., Armonk, NY, USA). Statistical significance was set at *p* < 0.05.

## Results

### Demographic and Clinical Characteristics

Four children underwent bilateral cochlear implantation, and all other children underwent unilateral cochlear implantation. Except for CND33, all participants were implanted with a contour electrode array, either a 24RE[CA] or CI512, in the test ear. CND33 underwent bilateral cochlear implantation and was implanted with the CI422 in the right ear and CI512 in the left ear. The electrode arrays were fully inserted in each ear. The participants' age at implantation ranged from 0.9 to 7.9 (mean: 2.8; standard deviation [SD]: 1.6) years. The test age ranged from 1.9 to 12.1 (mean: 4.9; SD: 2.6) years. All participants had normal FN function on implanted sides. In addition, no participants exhibited severe developmental delay or genetic-related hearing loss syndrome. The detailed demographic characteristics of the participants are shown in Table 1.

**Table 1:** Demographic information, imaging results and tested electrodes for each participant.

**Number**	**Gender**	**Ear tested**	**Age at implantation**	**Age at testing**	**Electrode array**	**Width of the BCNC (mm)**	**Diameter of VCN nerve (mm)**	**Diameter of facial nerve (mm)**	**Width of the IAC (mm)**	**Electrodes with ECAP**	**Tested electrodes**
CND1	F	L	3.36	5.4	24RECA	0.37	1.94	1.73	3.66	1–8	3, 10, 18
CND2	F	L	0.94	2.4	24RECA	0.57	1.9	1.86	3.98	0	None
CND3	M	L	2.81	5.5	24RECA	0.9	1.86	1.48	4.53	1–5, 19–21	1, 4, 21
CND4	F	R	1.55	4.0	24RECA	1.11	1.7	1.7	4.82	1–22	3, 12, 21
CND5	F	R	1.95	3.4	24RECA	0.8	0.99	1.36	1.96	9–10, 17–19, 21	9, 18, 21
CND6	F	R	1.30	2.6	24RECA	0.75	1.62	1.8	1.59	0	None
CND7	M	R	1.86	2.3	24RECA	0.77	0.9	0.5	3.1	1–22	3, 12, 21
CND7	M	L	1.86	2.4	24RECA	0.69	1.6	1.4	3	1–15	1, 7, 14
CND8	M	R	2.10	4.1	24RECA	0.63	1.64	1.86	2.5	1–8	1, 5, 8
CND9	M	L	1.24	2.3	24RECA	0.68	1.6	1	5.24	1–22	3, 12, 21
CND10	F	L	1.31	3.8	24RECA	0.93	1.1	1.3	5.53	1–7	1, 4, 7
CND11	F	L	5.95	8.0	24RECA	0	1.45	1.42	2.96	1–22	3, 12, 21
CND12	F	R	2.36	6.5	24RECA	0.82	2.08	1.35	4.83	1–22	3, 12, 21
CND13	M	L	5.60	11.2	24RECA	0	1.6	1.1	4.34	1–22	3, 12, 21
CND14	M	R	1.34	2.2	24RECA	0.56	1.32	1.51	3.54	1–22	3, 12, 21
CND14	M	L	1.34	2.0	24RECA	0.38	1.54	1.99	3.68	1–22	3, 12, 21
CND15	F	R	4.01	6.9	24RECA	1.04	1.1	0.9	3.96	1–22	3, 12, 21
CND16	F	L	4.44	8.1	24RECA	1.62	0.81	1.05	1.48	1–7	1, 3, 7
CND17	F	L	4.65	6.5	24RECA	0.97	1.53	1.87	3.48	1–22	3, 12, 21
CND18	M	L	7.93	12.1	24RECA	0.83	1.02	1.24	2.65	1–17	3, 10, 17
CND19	M	L	3.85	6.1	24RECA	0.77	1.7	1.5	3.81	1–7	1, 4, 7
CND20	M	R	1.93	4.1	24RECA	0.77	1.57	1.42	5.35	1–22	3, 12, 21
CND21	F	R	1.40	4.1	24RECA	1.07	1.3	1.7	5.22	1–9	1, 5, 9
CND22	M	R	2.61	6.8	24RECA	1.07	1.15	1.52	2.45	1–22	3, 12, 21
CND23	F	L	4.06	7.3	24RECA	0.53	1.6	1.64	4.73	1–8	1, 4, 8
CND24	F	R	1.20	2.2	24RECA	0.7	1.43	1.56	3.66	1–8	1, 4, 8
CND25	F	L	2.57	4.3	24RECA	0.76	1.65	1.51	3.04	1–11	1, 5, 11
CND26	F	R	2.93	8.5	24RECA	0.72	2.17	1.54	3.69	1–8	1, 4, 8
CND27	F	R	1.52	4.0	24RECA	0.91	1.22	0.57	3.2	1–22	3, 12, 21
CND27	F	L	1.52	2.1	24RECA	1.1	0.5	0.72	3.16	1–22	3, 12, 21
CND28	F	R	2.98	4.1	24RECA	0.7	1.7	1.4	3.05	1–22	3, 12, 21
CND29	M	L	1.94	2.5	24RECA	0.9	1.55	1.61	4.52	1–22	3, 12, 21
CND30	F	R	1.77	3.7	24RECA	0.69	1.42	1.7	4.02	1–22	3, 12, 21
CND31	F	R	1.91	3.4	24RECA	0.8	2.05	1.57	3.6	1–22	3, 12, 21
CND32	F	R	4.64	7.8	24RECA	0.87	VCN aplasia		1.86	1–22	3, 12, 21
CND33	F	R	2.63	3.4	CI422	0.9	VCN aplasia		2	1–22	3, 12, 21
CND33	F	L	2.63	3.7	CI512	0.8	1.5	0.8	1.95	1–15	3, 9, 15
CND34	F	L	3.06	4.2	24RECA	0.6	1.2	1.54	2.8	1–22	3, 12, 21
CND35	F	L	1.36	1.9	24RECA	0.72	0.45	0.3	2.36	1–22	3, 12, 21

### Imaging Results

Imaging findings for the individual participants are shown in Table 1. All tested ears, except for one, had a BCNC width <1.5 mm. BCNC atresia was observed in two ears, for which the width was defined as 0 mm. The mean BCNC width was 0.76 (SD: 0.28; range: 0–1.62) mm. On MRI, the cochlear nerve was absent on the reconstructed scans traversing the IAC in a perpendicular orientation and any plane of axial T2W sequence in all tested ears. All tested ears except for two had two nerve bundles, namely, the VCN and FNs, in the IAC on axial MRI imaging. The mean diameter of the VCN was 1.46 (SD: 0.36; range: 0.45–2.17) mm, and the mean diameter of the FN was 1.45 (SD: 0.40; range: 0.30–1.99) mm. The diameter of the VCN was smaller than that of the adjacent FN for 17 ears (44%), and the mean ratio of the VCN/FN diameter was 1.07 (SD: 0.32; range: 0.69–2.14). The ratio of the VCN/FN diameter was <1.5 in 31 ears (79%). In addition, the mean diameter of the IAC was 3.47 (SD: 1.10; range: 1.48–5.53) mm. Overall, 12 ears (31%) had IAC stenosis, with the diameter of the IAC ranging from 1.48 to 2.96 mm, while the other 27 ears (69%) had a normal IAC. In addition, five participants had Mondini malformation, and all the others had normal cochlear formation. Nine participants had vestibular or/and semicircular canal malformation, and all the others had normal vestibular and semicircular formation.

### Electrically Evoked Compound Action Potential Responses

**Figure 4:**
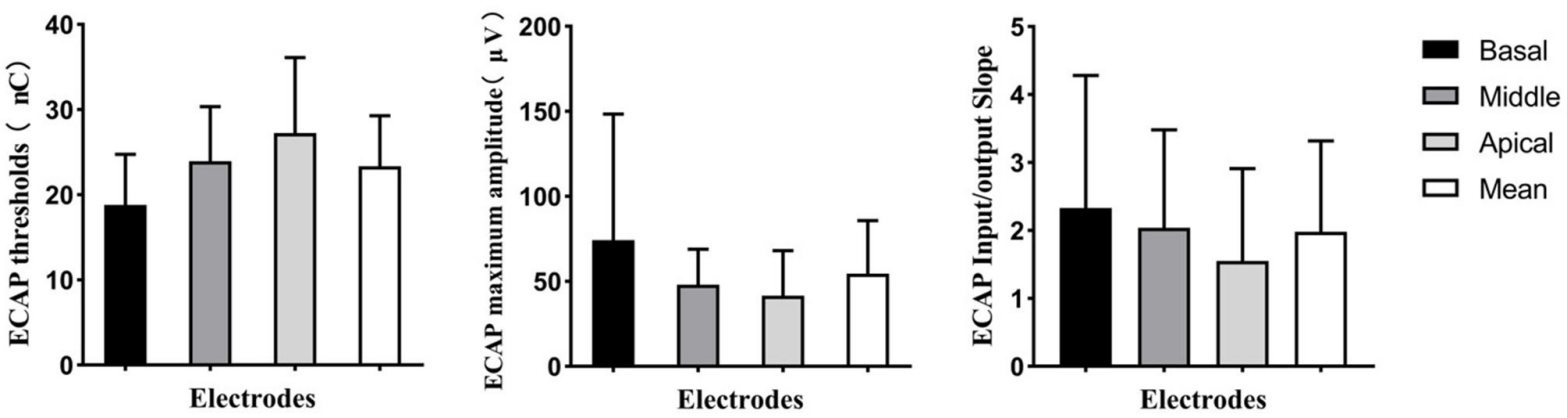
Means and standard deviations for ECAP threshold, maximum ECAP amplitude, and slope of the ECAP input/output function for basal, middle, and apical electrodes. The mean value of the ECAP at these three electrodes is also shown. ECAP, electrically evoked compound action potential.

Electrically evoked compound action potential responses were recorded at all activated electrodes in 22 ears (56%) but could not be recorded at any activated electrodes in two ears (6%). For the remaining 15 ears (38%), the ECAP response could only be recorded at some of the electrodes. The proportion of electrodes with measurable ECAP responses was 74.13%. Electrodes with ECAP responses and the tested electrodes for each participant are displayed in Table 1. Figure 4 shows the ECAP threshold, the maximum ECAP amplitude, and the slope of the ECAP I/O function at the three electrode locations and the mean values. At the basal, middle, and apical electrode locations, the mean ECAP thresholds were 18.80 (SD: 5.95; range: 10.50–36.77) nC, 23.96 (SD: 6.40; range: 13.28–38.32) nC, and 27.26 (SD: 8.58; range: 11.71–47.74) nC, respectively; the mean maximum ECAP amplitudes of ECAP were 74.28 (SD: 74.13; range: 9.73–456.45) μV, 48.02 (SD: 20.95; range: 106.56–24.08) μV, and 41.64 (SD: 26.52; range: 146.52–8.2) μV, respectively; and the mean slopes of ECAP I/O function were 2.33 (SD: 1.94; range: 8.70–0.11), 2.04 (SD: 1.44; range: 6.46–0.02), and 1.55 (SD: 1.36; range: 5.03–0.01), respectively. The ECAP results for each electrode are included in the Supplementary Material 1. As the electrode location moved from the basal to apical direction, the ECAP thresholds gradually increased and the maximum amplitudes and slopes of the ECAP I/O function gradually decreased. The repeated-measures ANOVA indicated that the electrode location had a significant effect on the ECAP thresholds (*F* = 30.34, *p* < 0.01), the maximum ECAP amplitude (*F* = 5.91, *p* < 0.01), and the slope of ECAP I/O function (*F* = 4.95, *p* = 0.01). When analyses were performed according to the participant, the mean ECAP threshold was 23.34 (SD: 5.93; range: 12.55–40.70) nC, mean maximum amplitude was 54.65 (SD: 31.04; range: 14.86–133.87) μV, and the mean slope of ECAP I/O function was 1.98 (SD: 1.34; range: 0.29–5.65).

### Relationship Between Imaging Results and ECAP Responses

Table 2 shows the relationships of the BCNC width, VCN diameter, and VCN/FN ratio with the ECAP threshold, the ECAP maximum amplitude, and the slope of the ECAP I/O function. BCNC width was not significantly correlated with the slope of the ECAP I/O function (*r* = −0.03, *p* > 0.05), the maximum amplitude (*r* = −0.06, *p* > 0.05), or ECAP thresholds (*r* = 0.15, *p* > 0.05). There were also no significant correlations between the VCN diameter and the slope of the ECAP I/O function (*r* = −0.9, *p* > 0.05), the maximum amplitude (*r* = 0.08, *p* > 0.05), or ECAP thresholds (*r* = 0.09, *p* > 0.05). The VCN/FN ratio was significantly correlated with the slope of the ECAP I/O function (*r* = 0.65, *p* < 0.01) and the maximum amplitude (*r* = 0.61, *p* < 0.01) but not with the ECAP threshold (*r* = −0.74, *p* > 0.05).

**Table 2:** Correlation of bone cochlear nerve canal (BCNC) width, vestibulocochlear nerve (VCN) diameter and VCN to facial nerve (FN) ratio to ECAP threshold, ECAP maximum amplitude and slope of ECAP Input/Output function.

	**Width of BCNC (*n* = 37)**	**Diameter of VCN (*n* = 35)**	**VCN/FN ratio** **(*n* = 35)**
ECAP thresholds	r = 0.15 *P* = 0.36	r = 0.09 *P* = 0.59	r = −0.07 *P* = 0.67
ECAP maximum amplitude	r = −0.06 *P* = 0.72	r = −0.08 *P* = 0.96	r = 0.61^**^ P < 0.01
Slope of ECAP input/output function	r = −0.03 *P* = 0.85	r = −0.09 *P* = 0.58	r = 0.65^**^ P < 0.01

** *presents p < 0.01*.

## Discussion

We first aimed to investigate cochlear nerve function using ECAP responses in individual children diagnosed with cochlear nerve aplasia based on imaging. In this study, the ECAP response was recorded in all but two participants. In participants with measurable ECAP responses, the percentage of electrodes with ECAP, ECAP thresholds, maximum amplitude, and slopes of the ECAP I/O function varied greatly among individual children, highlighting the variability of cochlear nerve function in individuals with cochlear nerve aplasia. Such variability may contribute to the various outcomes of cochlear implantation observed in children with cochlear nerve aplasia (Birman et al., 2016; Ehrmann-Muller et al., 2018; Yousef et al., 2021). Furthermore, in our patients, the slope of the ECAP I/O function and the maximum ECAP amplitude tended to gradually decrease, while the ECAP threshold tended to gradually increase, from basal to apical electrodes. This finding demonstrates that the responses of the cochlear nerve to electrical stimulation gradually decreased as the electrode location moved to a more apical location. These results are consistent with those of previous studies, which indicated that the degree of CND gradually worsens from the basal to the apical part of the cochlea in patients with CND (He et al., 2018; Xu et al., 2020). These specific characteristics of the cochlear nerve response to electrical stimulation for children with imaging-diagnosed cochlear nerve aplasia differ from those observed in children with normal-sized cochlear nerves diagnosed by MRI images. Previous studies have demonstrated that the electrode location exerts no significant effect on ECAP responses and that variations in ECAP results are much smaller among children with normal-sized cochlear nerves based on MRI (He et al., 2018). However, it was challenging to evaluate the function of the cochlear nerve for participants with no ECAP response in this study. Of the two participants without ECAP responses, one experienced some improvement in the ability to detect sound, while the other developed some close-set speech discrimination ability. Therefore, the absence of an ECAP response does not indicate the absence of a neural response in children diagnosed with cochlear nerve aplasia based on imaging results. A previous study indicated that the small ECAP responses for children with CND may be contaminated by artifacts related to electrical stimulation (He et al., 2020). In this study, we excluded the two children without ECAP responses from the correlation analysis.

The second aim of this study was to evaluate the possibility of using preoperative imaging results to predict the functional status of the cochlear nerve in children with imaging-diagnosed cochlear nerve aplasia. Research has indicated that the width of the BCNC is significantly smaller in children with imaging-diagnosed cochlear nerve aplasia than in other children with SNHL (Purcell et al., 2015). In this study, all but one child had BCNC stenosis, which further confirms that BCNC stenosis is a positive indicator of CND. Our results are consistent with those of previous reports. One such report indicated that ~84% of ears with BCNC stenosis had a deficient cochlear nerve, while all ears with BCNC atresia had CND (Tahir et al., 2020). Although BCNC stenosis or atresia is used to diagnose CND, BCNC width was not significantly correlated with ECAP responses in our study, indicating that it cannot be used to predict the degree of CND. Several previous studies have reported worse CI outcomes among patients with BCNC stenosis than among patients with a normal BCNC, while these studies performed group comparisons between patients with BCNC stenosis (BCNC width <1.5 or 1.4 mm) and other groups (such as patients with a BCNC width >1.5 mm) (Kang et al., 2016, 2019; Chung et al., 2018). However, almost all participants in our study had BCNC. Whether patients with more severe BCNC stenosis perform worse postimplantation has seldom been reported. Chao et al. reported no significant relationship between BCNC width and auditory/speech performance in 10 children with CND (Chao et al., 2016). Furthermore, ECAP responses were recorded in participants with BCNC atresia in this study, which indicated that there should be some functional spiral ganglion neurons innervating the cochlea even in these patients. Previous studies have also reported that patients with BCNC atresia may experience some benefits with CIs (Warren et al., 2010; Tahir et al., 2020). Our results and those of previous studies indicate that the absence of the BCNC does not preclude the presence of the cochlear nerve. Thus, the collected results indicate that BCNC width is not related to cochlear nerve function in patients with BCNC stenosis who have been diagnosed with cochlear nerve aplasia based on imaging.

Furthermore, we investigated the influence of VCN size on the functional status of the cochlear nerve. Since the cochlear nerve could not be precisely assessed on MR images in children with imaging-diagnosed cochlear nerve aplasia, the size of the VCN was evaluated at the cerebellopontine angle on the axial plane of MRI. The diameter of the VCN tested in this study is significantly smaller than in other children with SNHL reported in previous studies (Nadol and Xu, 1992). Previous histological studies have indicated that the diameter of the VCN is significantly correlated with the number of spiral ganglion neurons (Nadol and Xu, 1992). In this study, there was no significant correlation between VCN diameter and ECAP responses, which indicates that the absolute size of the VCN does not predict cochlear nerve function in children with cochlear nerve aplasia. However, the VCN/FN ratio exhibited a significant correlation with the slope of the ECAP I/O function and ECAP maximum amplitude, indicating that the VCN diameter in relation to that of the FN may predict the functional status of the cochlear nerve. Previous histological studies have also highlighted great variability in the diameter of the VCN or FN in humans with normal hearing and those with hearing loss (Nadol and Xu, 1992; Nakamichi et al., 2013). Therefore, the diameter of the VCN is not suitable for predicting the number of residual spiral ganglion neurons. Herein, all participants had normal FN function, although the range for the diameter of the FN was large. We believe that the ratio of VCN to FN diameter can eliminate the influence of the variation in VCN diameter among patients. Previous studies have also investigated the relationship of the relative size of the VCN with CI outcomes in children with CND (Yamazaki et al., 2015; Chao et al., 2016; Han et al., 2019). Studies by Han et al. and Yamazaki et al. have demonstrated a significant correlation between the relative size of the VCN and Categories of Auditory Performance (CAP) scores. However, Chao et al. reported no significant correlation between relative VCN size and CAP scores. Further analysis showed that only 10 patients were included in Chao et al.'s study and the follow-up time was short, which would affect the differences in outcomes among patients. Overall, the relative size of the VCN may represent a sensitive indicator for predicting cochlear nerve function in children with imaging-diagnosed cochlear nerve aplasia.

### Limitations

The main limitation of this study is that only three electrodes were used for each participant. In theory, the average ECAP results of all electrodes in the cochlea should be considered when examining the function of the cochlear nerve in each participant. However, electrodes exhibiting ECAP responses among participants were inconsistent, and it was time-consuming to test each electrode. Therefore, three representative cochlear electrodes were selected for this study. In addition to our study, previous studies have demonstrated that deficiency of the cochlear nerve progresses as a gradual increase from the basal to the apical region of the cochlea in children with CND (He et al., 2018; Xu et al., 2020). Thus, the average ECAP results from the basal, middle, and apical electrodes can roughly represent the function of the cochlear nerve. These three representative electrode sites were also used to estimate the function of the cochlear nerve in a previous study (Skidmore et al., 2020). In addition, patients with cochlear nerve hypoplasia have not been included in this study. It remains unclear whether there are some correlations between the relative size of the VCN and cochlear nerve function in children with cochlear nerve hypoplasia.

## Conclusion

Children diagnosed with cochlear nerve aplasia based on MRI imaging exhibit variations in the functional status of the cochlear nerve. For children with cochlear nerve aplasia, the width of the BCNC does not predict cochlear nerve function, and the absence of the BCNC does not preclude the presence of the cochlear nerve. Compared with the absolute size of the VCN, the size of the VCN relative to the FN may represent an indicator for predicting the functional status of the cochlear nerve in children with imaging-diagnosed cochlear nerve aplasia. For these children, a larger VCN relative to the size of the FN may be associated with better CI outcomes.

## Data Availability Statement

The datasets presented in this study can be found in online repositories. The names of the repository/repositories and accession number(s) can be found in the article/Supplementary Material.

## Ethics Statement

The studies involving human participants were reviewed and approved by the Ethical Committee of Shandong Provincial ENT Hospital affiliated with Shandong University. Written informed consent to participate in this study was provided by the participants' legal guardian/next of kin.

## Author Contributions

XC participated in data collection and patient testing, prepared the initial draft of this manuscript, provided critical comments, and approved the final version of this manuscript. RW and JL participated in data analysis, provided critical comments, and approved the final version of this manuscript. ZF and HW provided critical comments and approved the final version of this manuscript. LX designed the study, participated in data collection and patient testing, and drafted and approved the final version of this manuscript. All authors contributed to the article and approved the submitted version.

## Funding

This study was supported by grants from the National Natural Science Foundation of China (Nos. 81800905 and 82071053) and the Natural Science Foundation of Shandong Province Grant (No. ZR2016QZ007).

## Conflict of Interest

The authors declare that the research was conducted in the absence of any commercial or financial relationships that could be construed as a potential conflict of interest.

## Publisher's Note

All claims expressed in this article are solely those of the authors and do not necessarily represent those of their affiliated organizations, or those of the publisher, the editors and the reviewers. Any product that may be evaluated in this article, or claim that may be made by its manufacturer, is not guaranteed or endorsed by the publisher.

## Supplementary Material

Supplementary Material 1 All the raw data for the ECAP responses are displayed in the Supplementary Material (S1).
