# Supplementary material for: Value of Preoperative Imaging Results in Predicting Cochlear Nerve Function in Children Diagnosed With Cochlear Nerve Aplasia Based on Imaging Results
Source: Front Neurosci. 2022 Jun 14;16:905244. doi: 10.3389/fnins.2022.905244 (PMC9237450; doi:10.3389/fnins.2022.905244)
Supplement: Supplementary Material 1 — All the raw data for the ECAP responses are displayed in the Supplementary Material (S1). [file Data_Sheet_1.docx]

| Number | ECAP Threshold (nC) | | | | Maximum amplitude (uv） | | | | ECAP slope | | | |
| --- | --- | --- | --- | --- | --- | --- | --- | --- | --- | --- | --- | --- |
|  | Basal | Middle | Apical | Mean | Basal | Middle | Apical | Mean | Basal | Middle | Apical | Mean |
| CND1 | 19.19 | 29.61 | 38.13 | 28.98 | 79.41 | 61.99 | 8.20 | 49.87 | 3.49 | 3.25 | 0.11 | 2.28 |
| CND2 |  |  |  |  |  |  |  |  |  |  |  |  |
| CND3 | 21.39 | 32.41 | 12.00 | 21.93 | 103.48 | 55.33 | 58.40 | 72.40 | 2.70 | 1.15 | 0.81 | 1.55 |
| CND4 | 14.12 | 26.57 | 29.85 | 23.51 | 56.87 | 27.15 | 43.03 | 42.35 | 1.66 | 1.51 | 1.86 | 1.68 |
| CND5 | 37.75 | 17.54 | 16.32 | 23.87 | 9.73 | 44.06 | 73.26 | 42.35 | 1.32 | 1.08 | 0.01 | 0.80 |
| CND6 |  |  |  |  |  |  |  |  |  |  |  |  |
| CND7 | 15.63 | 13.28 | 11.71 | 13.54 | 123.29 | 106.56 | 8.20 | 79.35 | 8.70 | 6.46 | 1.27 | 5.48 |
| CND7 | 15.45 | 24.72 | 34.21 | 24.79 | 148.05 | 38.93 | 41.49 | 76.16 | 3.59 | 1.94 | 1.10 | 2.21 |
| CND8 | 10.02 | 13.14 | 17.22 | 13.46 | 108.61 | 38.42 | 30.74 | 59.26 | 3.46 | 1.81 | 1.37 | 2.21 |
| CND9 | 19.19 | 25.17 | 26.09 | 23.48 | 84.02 | 90.16 | 85.55 | 86.58 | 2.83 | 3.62 | 4.46 | 3.64 |
| CND10 | 18.18 | 23.84 | 30.15 | 24.06 | 55.33 | 42.52 | 11.27 | 36.37 | 0.87 | 0.96 | 0.30 | 0.71 |
| CND11 | 19.90 | 22.18 | 24.27 | 22.12 | 49.69 | 34.32 | 26.13 | 36.71 | 1.79 | 0.71 | 0.29 | 0.93 |
| CND12 | 18.18 | 22.58 | 30.15 | 23.64 | 42.01 | 30.74 | 28.18 | 33.64 | 2.01 | 1.94 | 0.89 | 1.61 |
| CND13 | 18.85 | 26.09 | 26.09 | 23.68 | 98.87 | 73.26 | 20.49 | 64.21 | 2.86 | 3.49 | 0.20 | 2.18 |
| CND14 | 15.18 | 19.90 | 22.58 | 19.22 | 21.00 | 32.79 | 35.86 | 29.88 | 0.47 | 0.73 | 1.02 | 0.74 |
| CND14 | 10.50 | 14.80 | 12.36 | 12.55 | 60.45 | 48.84 | 29.03 | 46.11 | 1.84 | 2.27 | 0.76 | 1.62 |
| CND15 | 16.32 | 22.58 | 28.56 | 22.49 | 45.08 | 60.45 | 28.17 | 44.57 | 2.32 | 2.43 | 1.60 | 2.12 |
| CND16 | 18.51 | 24.72 | 34.83 | 26.02 | 65.57 | 40.98 | 25.61 | 44.05 | 2.05 | 1.04 | 1.08 | 1.39 |
| CND17 | 21.01 | 23.84 | 29.08 | 24.64 | 22.54 | 29.20 | 39.45 | 30.40 | 0.24 | 0.12 | 2.36 | 0.91 |
| CND18 | 23.84 | 26.09 | 30.15 | 26.69 | 53.77 | 66.08 | 44.57 | 54.81 | 1.80 | 3.04 | 1.00 | 1.95 |
| CND19 | 36.77 | 38.82 | 46.50 | 40.70 | 26.64 | 24.08 | 16.91 | 22.54 | 2.03 | 1.34 | 1.06 | 1.48 |
| CND20 | 18.85 | 23.84 | 21.01 | 21.23 | 34.84 | 33.81 | 35.86 | 34.84 | 1.26 | 2.12 | 0.47 | 1.28 |
| CND21 | 19.90 | 21.78 | 31.26 | 24.31 | 34.32 | 33.81 | 26.64 | 31.59 | 0.56 | 0.65 | 0.20 | 0.47 |
| CND22 | 16.02 | 16.02 | 14.64 | 15.56 | 35.86 | 31.76 | 22.54 | 30.05 | 0.41 | 0.87 | 0.31 | 0.53 |
| CND23 | 24.92 | 36.41 | 47.74 | 36.36 | 64.55 | 49.18 | 33.30 | 49.01 | 1.12 | 1.57 | 1.39 | 1.36 |
| CND24 | 12.67 | 20.26 | 26.09 | 19.68 | 111.17 | 37.91 | 51.74 | 66.94 | 2.79 | 2.17 | 4.06 | 3.01 |
| CND25 | 23.41 | 30.70 | 35.47 | 29.86 | 126.03 | 62.05 | 36.37 | 74.82 | 6.22 | 4.07 | 1.73 | 4.01 |
| CND26 | 14.91 | 17.22 | 22.18 | 18.10 | 72.75 | 40.47 | 56.35 | 56.52 | 2.33 | 1.33 | 2.35 | 2.00 |
| CND27 | 16.75 | 22.77 | 26.31 | 21.94 | 23.05 | 25.10 | 40.47 | 29.54 | 0.25 | 0.60 | 1.19 | 0.68 |
| CND27 | 21.01 | 36.12 | 29.08 | 28.73 | 44.57 | 33.30 | 39.45 | 39.11 | 1.19 | 1.19 | 1.40 | 1.26 |
| CND28 | 20.26 | 30.70 | 31.83 | 27.60 | 35.86 | 48.67 | 47.64 | 44.06 | 0.99 | 1.90 | 2.13 | 1.67 |
| CND29 | 18.85 | 30.70 | 37.44 | 29.00 | 73.26 | 60.45 | 28.18 | 53.96 | 4.37 | 3.43 | 3.17 | 3.66 |
| CND30 | 14.20 | 15.83 | 20.02 | 16.68 | 52.94 | 80.94 | 89.19 | 74.36 | 1.73 | 1.96 | 3.31 | 2.33 |
| CND31 | 16.02 | 22.18 | 28.56 | 22.25 | 40.47 | 33.30 | 50.21 | 41.33 | 0.94 | 1.74 | 2.37 | 1.68 |
| CND32 | 11.37 | 17.54 | 23.41 | 17.44 | 153.18 | 90.68 | 146.52 | 130.13 | 6.30 | 5.86 | 4.79 | 5.65 |
| CND33 | 28.79 | 30.95 | 35.76 | 31.83 | 18.44 | 15.37 | 30.74 | 21.52 | 0.28 | 0.04 | 0.03 | 0.12 |
| CND33 | 16.32 | 19.90 | 21.78 | 19.33 | 456.45 | 63.01 | 36.88 | 185.45 | 6.08 | 3.18 | 1.77 | 3.68 |
| CND34 | 16.02 | 21.01 | 23.41 | 20.15 | 48.15 | 29.20 | 29.71 | 35.69 | 0.11 | 0.56 | 0.21 | 0.29 |
| CND35 | 15.18 | 24.72 | 32.41 | 24.10 | 68.14 | 61.99 | 84.53 | 71.55 | 3.29 | 3.41 | 5.05 | 3.92 |
